# Progression likelihood score identifies substages of presymptomatic type 1 diabetes in childhood public health screening

**DOI:** 10.1007/s00125-022-05780-9

**Published:** 2022-08-27

**Authors:** Andreas Weiss, Jose Zapardiel-Gonzalo, Franziska Voss, Manja Jolink, Joanna Stock, Florian Haupt, Kerstin Kick, Tiziana Welzhofer, Anja Heublein, Christiane Winkler, Peter Achenbach, Anette-Gabriele Ziegler, Ezio Bonifacio, for the Fr1da-study group

**Affiliations:** 1 Institute of Diabetes Research, Helmholtz Munich, German Research Center for Environmental Health, Munich, Germany; 2 German Center for Diabetes Research (DZD), Munich, Germany; 3 Forschergruppe Diabetes e.V. at Helmholtz Zentrum München, Munich, Germany; 4 Technical University Munich, School of Medicine, Forschergruppe Diabetes at Klinikum rechts der Isar, Munich, Germany; 5 Center for Regenerative Therapies Dresden, Faculty of Medicine, Technische Universität Dresden, Dresden, Germany; 6 Paul Langerhans Institute Dresden of Helmholtz Centre Munich at University Clinic Carl Gustav Carus of TU Dresden, Faculty of Medicine, Dresden, Germany

**Keywords:** Clinical trial modelling, Glucose tolerance, Immunotherapy, Islet autoantibodies, Population screening, Progression score, Public health screening, Type 1 diabetes

## Abstract

**Aims/hypothesis:**

The aim of this study was to develop strategies that identify children from the general population who have late-stage presymptomatic type 1 diabetes and may, therefore, benefit from immune intervention.

**Methods:**

We tested children from Bavaria, Germany, aged 1.75–10 years, enrolled in the Fr1da public health screening programme for islet autoantibodies (*n*=154,462). OGTT and HbA_1c_ were assessed in children with multiple islet autoantibodies for diagnosis of presymptomatic stage 1 (normoglycaemia) or stage 2 (dysglycaemia) type 1 diabetes. Cox proportional hazards and penalised logistic regression of autoantibody, genetic, metabolic and demographic information were used to develop a progression likelihood score to identify children with stage 1 type 1 diabetes who progressed to stage 3 (clinical) type 1 diabetes within 2 years.

**Results:**

Of 447 children with multiple islet autoantibodies, 364 (81.4%) were staged. Undiagnosed stage 3 type 1 diabetes, presymptomatic stage 2, and stage 1 type 1 diabetes were detected in 41 (0.027% of screened children), 30 (0.019%) and 293 (0.19%) children, respectively. The 2 year risk for progression to stage 3 type 1 diabetes was 48% (95% CI 34, 58) in children with stage 2 type 1 diabetes (annualised risk, 28%). HbA_1c_, islet antigen-2 autoantibody positivity and titre, and the 90 min OGTT value were predictors of progression in children with stage 1 type 1 diabetes. The derived progression likelihood score identified substages corresponding to ≤90th centile (stage 1a, *n*=258) and >90th centile (stage 1b, *n*=29; 0.019%) of stage 1 children with a 4.1% (95% CI 1.4, 6.7) and 46% (95% CI 21, 63) 2 year risk of progressing to stage 3 type 1 diabetes, respectively.

**Conclusions/interpretation:**

Public health screening for islet autoantibodies found 0.027% of children to have undiagnosed clinical type 1 diabetes and 0.038% to have undiagnosed presymptomatic stage 2 or stage 1b type 1 diabetes, with 50% risk to develop clinical type 1 diabetes within 2 years.

**Graphical abstract:**

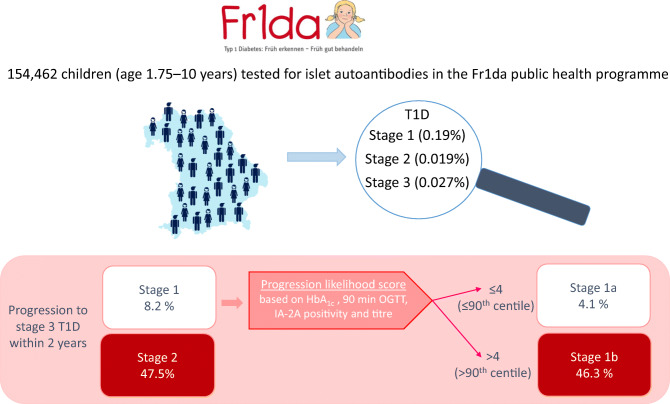

**Supplementary Information:**

The online version contains peer-reviewed and unedited supplementary material available at 10.1007/s00125-022-05780-9.

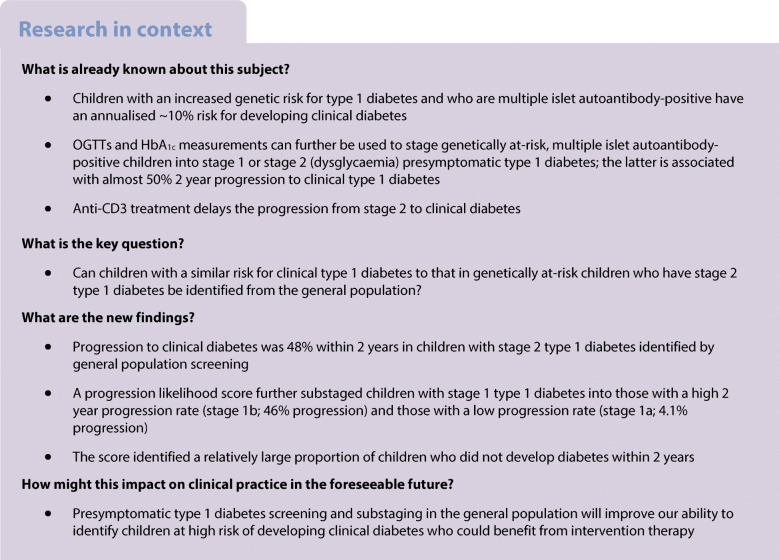



## Introduction

Type 1 diabetes can be delayed by immunotherapy in individuals with late-stage (stage 2) presymptomatic type 1 diabetes [1, 2]. This finding should encourage attempts to find new strategies that can augment the effectiveness of immunotherapy. A challenge, however, is the relative infrequency of potential trial participants meeting the criteria for stage 2 type 1 diabetes in people with a first- or second-degree family history of type 1 diabetes. This leads to long trial recruitment, a setting that is unattractive for prevention studies [1]. Moreover, since most individuals do not have a family history of type 1 diabetes, the large majority who may benefit from treatment are excluded from trials. Potential avenues to relieve this challenge include widening the screening programmes beyond the genetically at-risk population [3] and identifying subgroups of individuals with stage 1 type 1 diabetes who progress to clinical diabetes at similar rates to individuals with stage 2 type 1 diabetes. Childhood population-based islet autoantibody screening is possible, as demonstrated by the Fr1da study [4]. Around 0.3% of German children have presymptomatic type 1 diabetes with a 9% annualised risk of progression to clinical (stage 3) diabetes, which approaches the risk observed in multiple islet autoantibody-positive genetically at-risk children [5, 6]. The objective of this study was to develop a substaging strategy that identifies children within a general population who are at high risk of developing clinical type 1 diabetes within 2 years.

## Methods

### Participants and measures

Between February 2015 and July 2021, children in Bavaria, Germany, with no previous diagnosis of diabetes were offered screening for islet autoantibodies by primary care paediatricians in the context of well-child visits [4]. Children aged 1.75–5.99 years were eligible until March 2019, and children aged 1.75–10.99 years were eligible from April 2019 to July 2021. A total of 154,462 children with a median age of 3.14 years (IQR 2.16–4.97 years) participated in the screening. This included 6099 (3.9%) who had a first-degree relative with type 1 diabetes. The study was approved by the institutional review board at Technical University Munich. Written informed consent was obtained from the children’s parents or legal guardians.

Islet autoantibody testing was performed on capillary blood using the 3Screen ELISA, which measures autoantibodies to glutamic acid decarboxylase (GADA), insulinoma antigen-2 (IA-2A) and zinc transporter 8 (ZnT8A) [7, 8]. Samples above a threshold of 25 U were tested for insulin autoantibodies (IAA), GADA, IA-2A and ZnT8A using radiobinding assays with radiolabelled human insulin, radiolabelled human GAD65 (amino acids 96-585), radiolabelled human IA-2 (amino acids 606-979) and radiolabelled 325 W and R variants of human ZnT8 (amino acids 268-369) as previously described [9–11]. A confirmatory sample was requested and tested in children positive for two or more of these autoantibodies. Presymptomatic type 1 diabetes was defined as positivity for two or more autoantibodies in both the screening sample and a confirmatory sample. Families of children with presymptomatic type 1 diabetes were invited to participate in metabolic staging and an educational programme at a paediatric diabetes clinic close to their residence. The results of an OGTT and HbA_1c_ were used for staging. Weight, height and BMI were also assessed. Genotyping for 46 SNPs was performed to calculate a genetic risk score (ESM Table 1) if consent for ancillary research was provided [12].

Children who were positive for multiple islet autoantibodies were classified as stage 1, stage 2 or stage 3 type 1 diabetes, corresponding to the consensus criteria of the JDRF, Endocrine Society and ADA of 2015, as previously described [13]. Stage 1 type 1 diabetes was defined as positivity for two or more islet autoantibodies and normal glucose tolerance based on the results of OGTT. Stage 2 was defined as two or more islet autoantibodies accompanied by dysglycaemia based on OGTT results (fasting plasma glucose of 6.1–6.9 mmol/l [110–125 mg/dl] or impaired 2 h plasma glucose of 7.8–11.0 mmol/l [140–199 mg/dl], and/or plasma glucose ≥ 11.1 mmol/l [200 mg/dl] at 30, 60 or 90 min). Stage 3 type 1 diabetes was defined based on ADA criteria: fasting plasma glucose ≥7.0 mmol/l (126 mg/dl) or a 2 h plasma glucose of ≥11.1 mmol/l (200 mg/dl) in an OGTT; or HbA_1c_ >48 mmol/mol (6.5%); or in children with classic symptoms of hyperglycaemia, a random plasma glucose of >11.1 mmol/l (200 mg/dl) in the absence of unequivocal hyperglycaemia. The first three criteria required confirmation by repeat testing. Revised stage 2 ADA criteria have been released (impaired fasting plasma glucose 5.6–6.9 mmol/l [100–125 mg/dl] and/or impaired glucose tolerance 2 h plasma glucose 7.8–11.0 mmol/l [140–199 mg/dl] and/or HbA_1c_ 39–47 mmol/mol [5.7–6.4%] or ≥10% increase in HbA_1c_) [14]), and an alternate analysis using these criteria is also presented. Children were monitored at intervals of 2–6 months for OGTT, HbA_1c_ and symptoms [4]. The last follow-up date for this analysis was 29 November 2021. Families of children who withdrew from the study or refused OGTTs were contacted by telephone and asked if the child had developed diabetes. Children with stage 1 type 1 diabetes were asked to participate in a mechanistic intervention study (ClinTrials.gov registration no. NCT02620072), with 1:1 randomisation to treatment with oral insulin or placebo for 12 months.

### Outcome definition

The primary outcome for this analysis was clinical (stage 3) type 1 diabetes.

### Statistical analyses

The progression to clinical diabetes was assessed using the Kaplan–Meier time-to-event method. Children were censored when they developed stage 3 type 1 diabetes or reached the date of their final contact to ascertain diabetes status. Between-group comparisons in the Kaplan–Meier analyses were performed using the logrank test. The Cox proportional hazards (CPH) model and logistic regression (LR) were used to identify factors that were associated with progression to stage 3 type 1 diabetes within 2 years. Prior to analysis, the BMI was transformed to a standardised BMI based on SD scores using WHO reference values [15].

Overweight status was defined as standardised BMI of 1–2 and obesity as BMI >2, according to WHO recommendations. For the CPH analysis, all variables were first analysed in univariable models. Variables with a two-tailed α<0.05 representing distinct parameters were included in a multivariable model. Variables that remained significant (*p*<0.05) were included in the progression likelihood score based on their estimates derived from the multivariable CPH. For the LR analysis, the significant variables were identified based on their importance in both a penalised LR model and a random forest model, each built independently using all variables. The outcome was a categorical variable encoding the progression to stage 3 type 1 diabetes within 2 years of follow-up. Tenfold cross-validation was used to select the optimal parameters for the LR and CPH models. The shared, most important variables in both models were included in an LR model and the estimates were used to calculate the progression likelihood score. The LR and random forest analyses were performed using R version 4.0.2 and the packages ‘tidymodels’ v0.1.2 [16], ‘themis’ v0.1.3 [17] and ‘vip’ v0.3.2 [18]. The survival analyses and plots were prepared using the package ‘survival’ v3.2-7 [19].

## Results

**Fig. 1:**
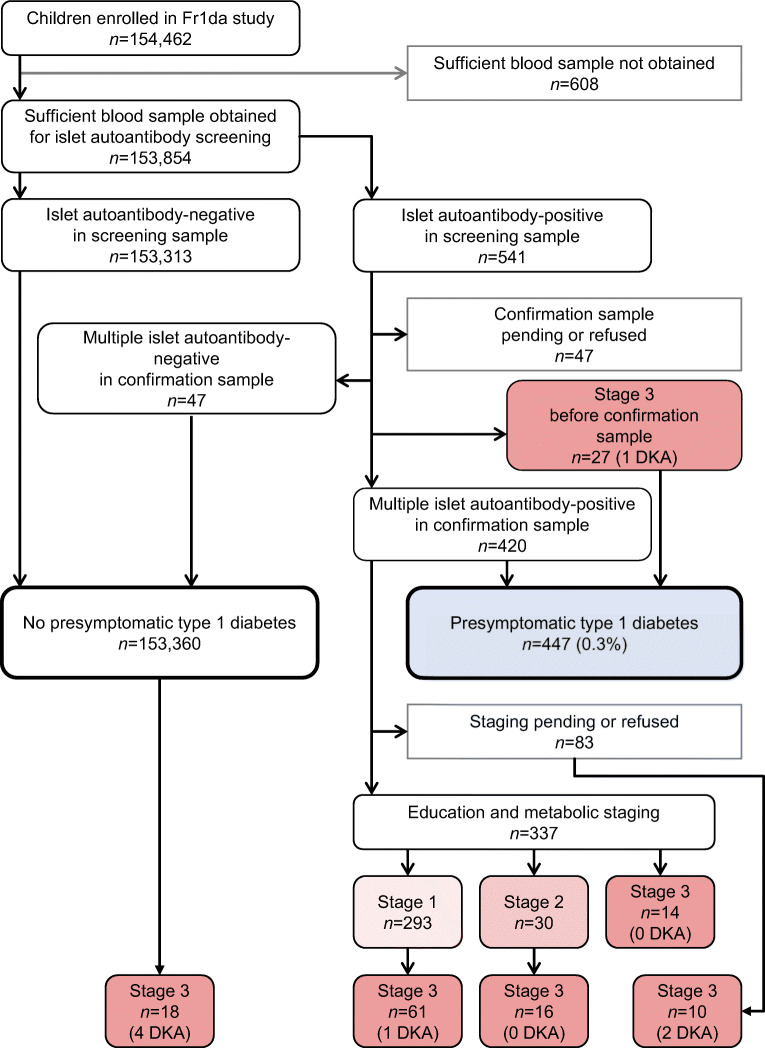
Flow of participants in the Fr1da study. DKA, diabetic ketoacidosis

Of 154,462 children screened, 447 (0.29% [95% CI 0.26, 0.32]) were diagnosed with multiple islet autoantibodies (presymptomatic type 1 diabetes, median age of 4.1 years [IQR 3.1–5.4 years]). Of these 447 children, 364 could be staged (Fig. 1). Of these, 293 children (representing 0.19% of all screened children) were diagnosed with stage 1 type 1 diabetes. Thirty children (representing 0.019% of all screened children) were diagnosed with stage 2 type 1 diabetes. Forty-one children were diagnosed with stage 3 type 1 diabetes, representing 0.027% of screened children. Of the 293 children with stage 1 type 1 diabetes, 149 (51%) participated in the mechanistic intervention study [4]. Unstaged children had similar characteristics to those who underwent follow-up (ESM Table 2).

### Progression to stage 3 type 1 diabetes

**Fig. 2:**
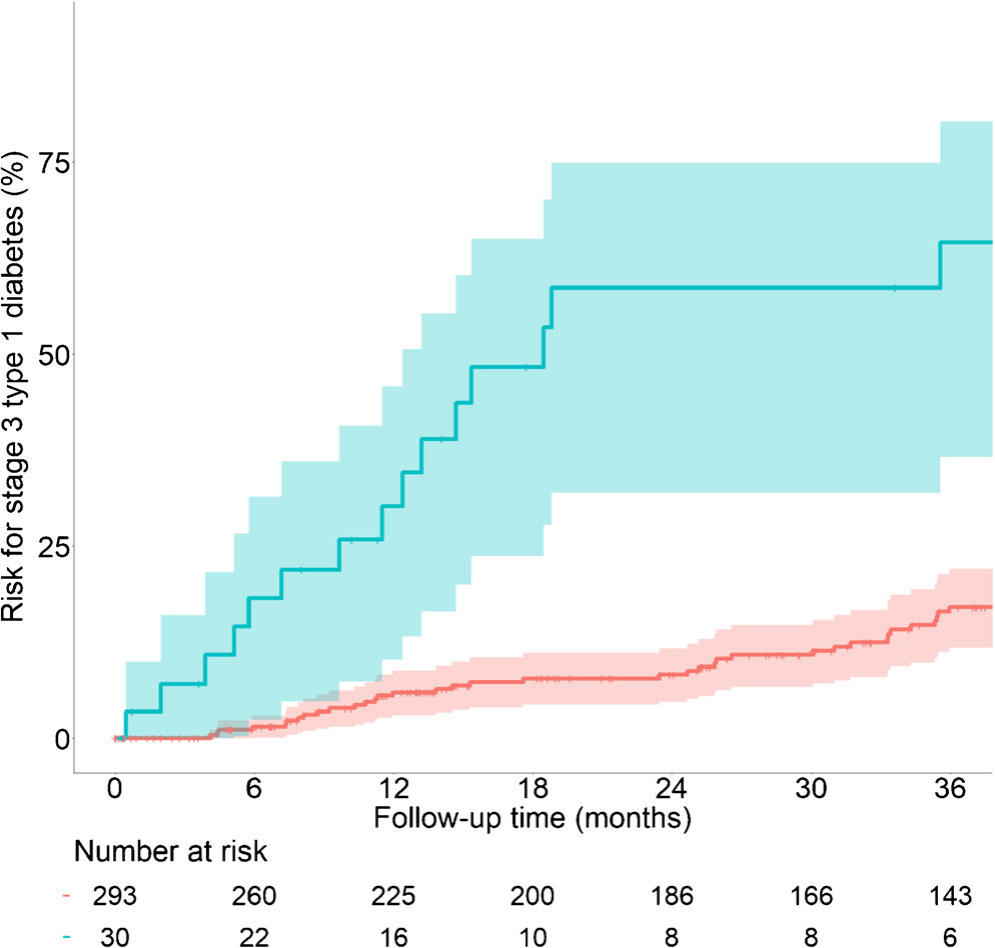
Progression from stage 1 or stage 2 type 1 diabetes to stage 3 type 1 diabetes. Cumulative risks (lines with 95% CIs indicated with shading) of developing stage 3 type 1 diabetes in children with stage 1 (red) or stage 2 (blue) type 1 diabetes. The follow-up starts at the initial staging using OGTTs. The numbers of children at risk are indicated below each time point

During the follow-up, an additional 77 children initially staged as stage 1 or stage 2 type 1 diabetes progressed to stage 3 type 1 diabetes (Fig. 1). The 2 year risk for progression to stage 3 type 1 diabetes was 58.6% (95% CI 32.0, 74.9) in the 30 children with stage 2 type 1 diabetes compared with 8.2% (95% CI 4.7, 11.6) in the 293 children with stage 1 type 1 diabetes (*p*<0.0001; Fig. 2). During the follow-up of children with presymptomatic type 1 diabetes (median [IQR] time 2.8 [1.0–4.6] years), 57 progressed to stage 2 type 1 diabetes. Overall, the 2 year risk of progression to stage 3 type 1 diabetes in the 87 children with stage 2 type 1 diabetes was 48% (95% CI 34, 58), with an annualised risk of 28% (see electronic supplementary material [ESM] Fig. 1).

### Two-year risk progression likelihood scores in stage 1 type 1 diabetes

**Fig. 3:**
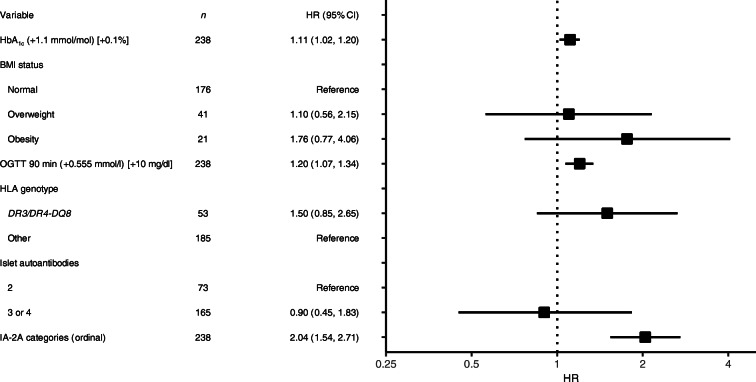
Multivariable analysis of risk of developing stage 3 type 1 diabetes. The HRs (95% CIs) are shown for variables included in the multivariable CPH analysis that were significantly associated with the development of stage 3 type 1 diabetes in univariable CPH models. IA-2A category as negative (0) and tertiles of IA-2A titres: 3–100 arbitrary units (1), 100–290 arbitrary units (2), >290 arbitrary units (3). Standardised BMI was calculated using the WHO Child Growth Standards based on height and weight and age

Factors that predicted progression to stage 3 type 1 diabetes in children with stage 1 type 1 diabetes were examined using CPH and LR analyses. In univariable CPH analyses, the predictive factors were HbA_1c_, glucose at each measurement time during OGTT, BMI, *HLA DR3/DR4-DQ8* genotype, IA-2A positivity and titre, and positivity for three or four islet autoantibodies (ESM Table 3). The multivariable CPH model selected IA-2A (adjusted HR per increase in ordinal value 2.04 [95% CI 1.54, 2.71]; *p*<0.0001), HbA_1c_ (adjusted HR per 1.1 mmol/mol [0.1%] increase 1.11 [95% CI 1.02, 1.20]; *p*=0.004) and glucose at 90 min during OGTT (adjusted HR per 0.555 mmol/l [10 mg/dl] increase 1.20 [95% CI 1.07, 1.34]; *p*<0.0001) as predictors of progression to clinical diabetes (Fig. 3).

LR analysis was performed for children with stage 1 type 1 diabetes who were followed for at least 2 years or who developed stage 3 type 1 diabetes within 2 years (*n*=202). Significant factors associated with 2 year progression, based on the variable’s importance in the penalised LR and random forest analyses, were IA-2A titre, HbA_1c_ and glucose at 90 min during OGTT (ESM Table 4, ESM Fig. 2). In both models, a likelihood of progression score was determined based on the parameter estimates for models that included the three significant variables:

$$ \mathrm{CPH}:\exp \left[\left({\mathrm{HbA}}_{1\mathrm{c}}-5.233\right)\times 1.125+\left({\mathrm{OGTT}}_{90}-107.6\right)\kern0.45em \times 0.0195+\left(\mathrm{IA}\hbox{-} {2\mathrm{A}}_{\mathrm{cat}}-1.27\right)\times 0.662\right)\left]\right[\mathrm{LR}:\kern0.95em 2.048\times {\mathrm{HbA}}_{1\mathrm{c}}+0.034\times {\mathrm{OGTT}}_{90}+0.006\times \mathrm{IA}\hbox{-} 2\mathrm{A} $$

where HbA_1c_ is measured in % and OGTT_90_ is measured in mg/dl.

**Fig. 4:**
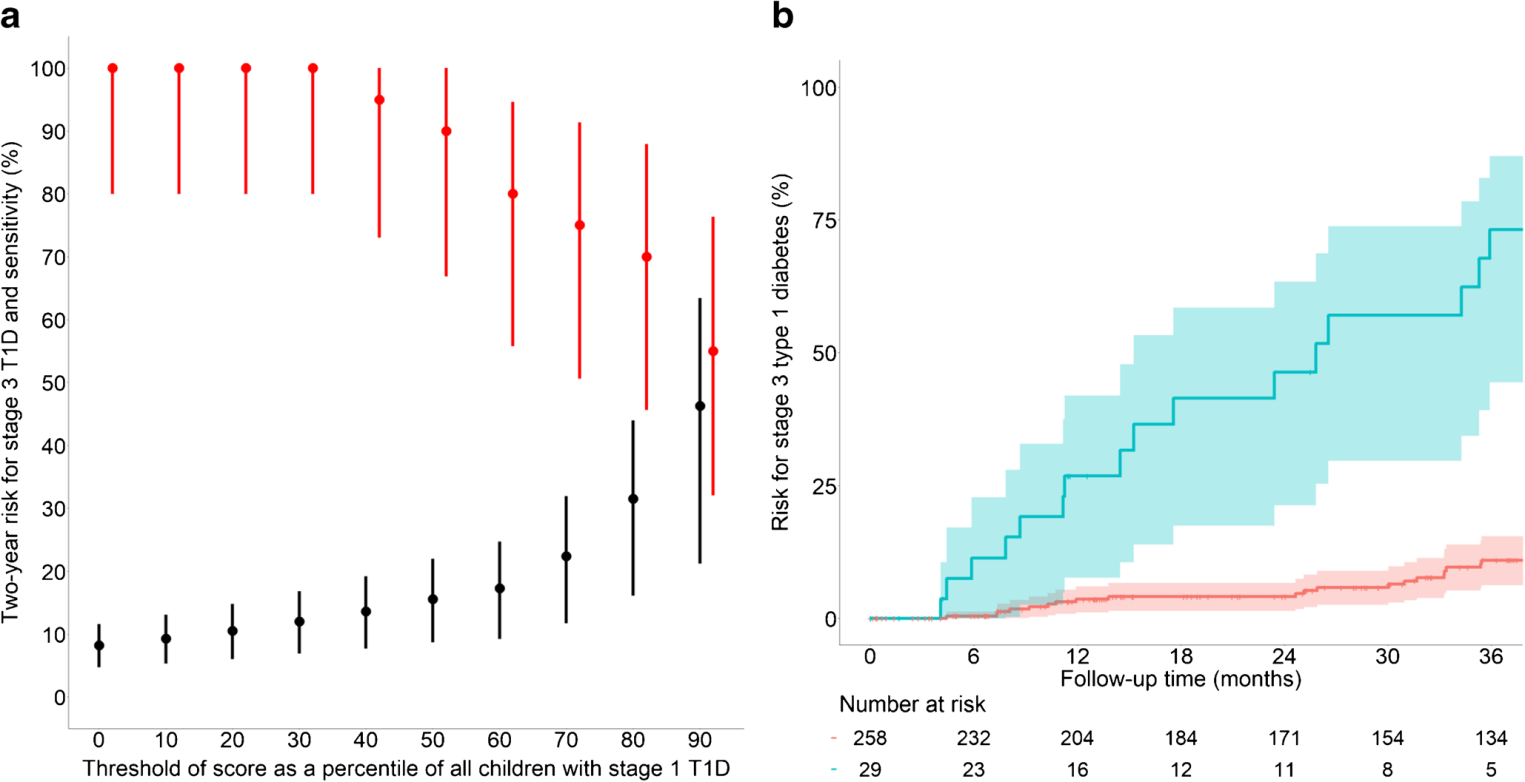
Progression to stage 3 type 1 diabetes in children with stage 1 type 1 diabetes stratified by the progression likelihood score. (**a**) Sensitivity (in red) and cumulative risk (in black) of developing stage 3 type 1 diabetes within 2 years in children with stage 1 type 1 diabetes, stratified by the tenth centiles of the risk scores calculated using Cox regression (progression likelihood score). Analyses were performed using 287 children with stage 1 type 1 diabetes and complete data for all three variables were included in the risk score (glucose at 90 min in OGTTs, IA-2A titre category and HbA_1c_). (**b**) Cumulative risks (lines with 95% CIs indicated with shading) of developing stage 3 type 1 diabetes in children with stage 1 type 1 diabetes and a risk score >90th centile (in blue) or ≤90th centile (in red). The follow-up starts at the initial staging. The numbers of children at risk are indicated below each time point. T1D, type 1 diabetes

The 2 year risks as well as the sensitivity for identifying children who progressed to stage 3 type 1 diabetes were determined for each tenth centile of the scores derived by the CPH (Fig. 4a) and LR (ESM Fig. 3) analyses. The threshold of score corresponding to the upper 90th centile (>4.0 for CPH; >17.1 for LR) yielded a 2 year risk of 46% (95% CI 21, 63) for the CPH model (Fig. 4b) and 45% (95% CI 21, 61) for the LR model (ESM Fig. 4). For both scores, 100,000 bootstrap replicates were performed to assess the accuracy and precision of both estimates (ESM Table 5). The sensitivity was 55% for both analyses. By comparison, the 2 year risk in children with scores ≤90th centile was 4.1% (95% CI 1.4, 6.7) for both the CPH and LR models (Fig. 4b and ESM Fig. 4). Neither the three factors individually nor a combination of the IA-2A and HbA_1c_ values were able to achieve comparable sensitivity and risk values (ESM Table 6). The sensitivity, specificity and positive predictive value of CPH score >4.0 to predict stage 3 type 1 diabetes within 2 years in the 202 children with stage 1 type 1 diabetes who had either reached the stage 3 type 1 diabetes outcome or had at least 2 years of follow-up were 55%, 94% and 50%, respectively. The sensitivity, specificity and positive predictive value of an LR score >17.1 to predict stage 3 type 1 diabetes within 2 years in the 202 children with stage 1 type 1 diabetes who had either reached the stage 3 type 1 diabetes outcome or had at least 2 years follow-up were 55%, 93% and 48%, respectively. These yield a likelihood ratio for a positive of 7.9, a likelihood ratio for a negative of 0.5 and a diagnostic OR of 17.3.

A validation cohort from the general screening population was unavailable. Therefore, we identified 46 children (median [IQR] age, 10.8 [8.1–13.4] years) with stage 1 type 1 diabetes and available data to calculate the progression likelihood score from German prospective studies of genetically at-risk children [20]. Three (6.5%) of these children had a CPH score >4.0 and the same three had an LR score >17.1. In total, 22 of the 46 children developed stage 3 type 1 diabetes, including four within 2 years of follow-up, and 18 within up to 9.6 years of follow-up. These children included two of the three with elevated progression likelihood scores (ESM Fig. 5).

### Subclassification of stage 1 type 1 diabetes

**Fig. 5:**
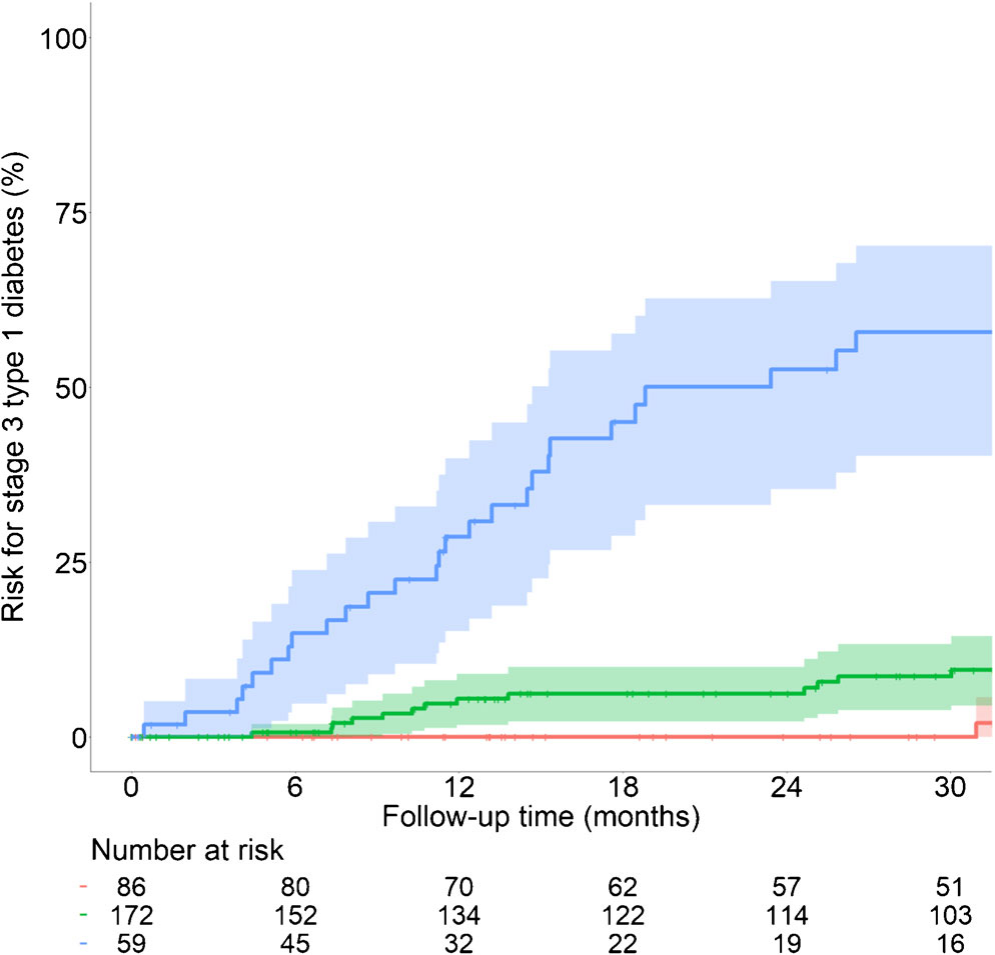
Progression to stage 3 type 1 diabetes in children with stage 2 or stage 1 type 1 diabetes stratified by the progression likelihood score. The cumulative risks (lines with 95% CIs indicated with shading) of developing stage 3 type 1 diabetes are shown for children with either stage 2 type 1 diabetes or stage 1b, corresponding to stage 1 type 1 diabetes, and a progression likelihood score >90th centile (in blue), children with stage 1a and a progression likelihood score between the 30th and 90th centiles (in green), and children with stage 1a and a progression likelihood score below the 30th centile (in red). The follow-up starts at the initial staging. The numbers of children at risk are indicated below each time point

The CPH model score was chosen to subclassify children into stage 1a (≤90th score centile) and stage 1b (>90th score centile) type 1 diabetes. Of 154,462 children screened for islet autoantibodies, 59 (0.038%) had either stage 1b (ESM Table 7) or stage 2 type 1 diabetes at initial staging. The 2 year risk of developing stage 3 type 1 diabetes in these 59 children was 52.6% (95% CI 35.4, 65.2), with an annualised risk of 31% (Fig. 5). Among the remaining 258 children with stage 1a type 1 diabetes, the risk could be further stratified by the progression likelihood score. None of the 86 children with stage 1a type 1 diabetes and scores <30th centile progressed to clinical diabetes within 2 years, and the 2 year risk was 6.2% (95% CI 2.2, 10.0; *p*=0.011) in the 172 children with scores between the 30th and 90th centiles. During follow-up, 26 (10%) children with stage 1a type 1 diabetes developed a progression likelihood score >4.0 (stage 1b type 1 diabetes) at a median follow-up time of 24.6 months (IQR 8.1–13.4). The 2 year risk of developing stage 3 type 1 diabetes in these children was 48.5% (95% CI 15.2, 68.8; ESM Fig. 6).

### Classification and progression according to revised ADA stage 1 and stage 2 type 1 diabetes criteria

The ADA has introduced modified criteria for stage 1 and stage 2 presymptomatic type 1 diabetes [14]. According to these criteria, 284 of the Fr1da study children would be diagnosed with stage 1 type 1 diabetes and 39 children would be diagnosed with stage 2 type 1 diabetes. Progression to stage 3 type 1 diabetes within 2 years was 42.5% (95% CI 22.5, 57.4) in the 39 children with ADA stage 2 type 1 diabetes and 8.5% (95% CI 4.8, 12) in children with ADA stage 1 type 1 diabetes. Of the 284 children diagnosed with ADA stage 1 type 1 diabetes, 256 met the criteria for stage 1a type 1 diabetes and 28 met the criteria for stage 1b type 1 diabetes as defined by the CPH model. Progression to stage 3 type 1 diabetes within 2 years was 45.8% (95% CI 19, 63.7) in the children with stage 1b type 1 diabetes.

### Population-based recruitment potential and cost

Modelling these data to a 1:1 randomised controlled prevention trial with 2 year recruitment, an average follow-up of 2 years and a total trial duration of 3 years to detect a treatment effect of 50% reduction in risk (two-tailed α=0.05, 80% power, 15% dropout) requires the enrolment of 154 children. Based on a 50% participation rate, it would be necessary to screen approximately 810,000 children to identify 308 who were eligible with stage 1b or stage 2 type 1 diabetes at initial staging. The total cost of screening and staging in the Fr1da study was recently estimated to be €21.73 per child [21], resulting in a screening cost of €17.6 million for the trial. The screening of the 810,000 children would also be expected to identify a further 219 (0.027%) children with undiagnosed stage 3 type 1 diabetes. Therefore, the cost per child identified with stage 3 type 1 diabetes, or with a 50% 2 year risk of developing stage 3 type 1 diabetes, by such a screening would be €33,400.

## Discussion

Childhood population-based screening for islet autoantibodies, followed by metabolic staging, identified 0.065% of children with undiagnosed stage 3 type 1 diabetes (0.027% of screened) and children with a 50% risk of developing stage 3 type 1 diabetes within 2 years (0.038% of screened). This high-risk group comprised children with stage 2 type 1 diabetes or a subgroup of stage 1 type 1 diabetes (stage 1b type 1 diabetes) with normal glucose tolerance and an increased progression likelihood score. The progression likelihood score was also able to identify 30% of children with stage 1a type 1 diabetes who would remain free of diabetes for at least 2 years. These findings endorse the screening and staging of children from the general population for inclusion in type 1 diabetes prevention trials and stage-appropriate counselling.

The study is one of the largest of children with multiple islet autoantibodies and is representative of the general population because it involves children who participated in a public health screening programme and were not preselected based on genetic or familial diabetes risk. We also determined frequencies of stage 1a, stage 1b and stage 2 type 1 diabetes that would be identified by a general population autoantibody testing programme and are useful for designing immunotherapy studies and modelling the associated screening costs. Since 22% of children identified as multiple islet autoantibody positive without stage 3 type 1 diabetes were not followed, the actual population frequencies of stage 1a, stage 1b and stage 2 type 1 diabetes are likely to be slightly higher than the frequencies achieved by the programme. A limitation of the study is the lack of similar general population cohorts that allow the validation of the progression likelihood scores. Therefore, validation was performed by the assessment of scores in a second, smaller set of older children who were part of a prospectively followed genetically at-risk cohort. Although the numbers were small, the second dataset confirmed both the 2 year risk and the sensitivity of a progression likelihood score >90th centile. The score could also be applied robustly during follow-up to identify additional children at high risk of developing stage 3 type 1 diabetes. An important finding that indirectly validates the Fr1da cohort and methods used for staging in the cohort is that the risk of progression to stage 3 type 1 diabetes among children with stage 2 type 1 diabetes was remarkably similar to that observed in the TrialNet study of relatives of individuals with type 1 diabetes [1]. Moreover, the score was shown to be applicable both in 2015 and to the more recently defined staging criteria for stage 1 and stage 2 type 1 diabetes. However, the transferability of the progression likelihood score to other studies was not tested. Nevertheless, the score includes a harmonised measurement of IA-2A that has been compared among international laboratories [22] and metabolic variables that are regularly evaluated in quality assurance programmes, suggesting that the threshold of 4 for the CPH score and 17.1 for the LR score could be adopted in other studies. These findings were derived from children living in Bavaria, Germany, who are largely of European descent. The applicability of the findings to other ethnic groups or to older children and adolescents where teplizumab therapy is effective has not been tested. Another limitation is that almost 50% of the children enrolled in a mechanistic intervention study [4]. Participation in the mechanistic study, however, did not significantly affect progression (ESM Table 3) and the progression likelihood score was also effective in stratifying risk when applied only to children who did not participate in the intervention study (ESM Fig. 7).

We and others have shown the value of IA-2A, HbA_1c_ and intermediate OGTT time points in stratifying risk in islet autoantibody-positive individuals who are genetically at risk for type 1 diabetes [23–33]. Some have developed scores that can stratify risk in relatives with either stage 1 or stage 2 type 1 diabetes [31–33]. The calculation of these scores requires OGTT C-peptide concentrations, which were unavailable in the majority of the Fr1da cohort. We cannot exclude the possibility that the inclusion of C-peptide concentration in the progression likelihood score will improve stratification. However, the performance of the score suggests that C-peptide measurements are not required to identify very high-risk children. The genetic risk score did not discriminate progression in the univariable analysis, while the presence of *HLA DR3/DR4-DQ8* was associated with faster progression in the univariable analyses but not in the multivariable model. Type 1 diabetes genetic risk scores are helpful for identifying higher-risk single islet autoantibody-positive individuals but provide limited if any improvement in risk stratification once children have multiple islet autoantibodies [12, 34, 35]. *HLA DR3/DR4-DQ8* was associated with faster progression in a study of genetically at-risk multiple islet autoantibody-positive children [6]. The presence of HLA class I alleles A24 or B18 are associated with faster progression in multiple islet autoantibody-positive first-degree relatives of individuals with type 1 diabetes [36]. It is, therefore, possible that the inclusion of these and other genes could improve progression likelihood scores.

One objective of this study was to refine the strategies that accelerate the recruitment of high-risk children into clinical trials of late-stage presymptomatic type 1 diabetes through population-based islet autoantibody screening. We estimated that an appropriately powered trial of 154 children with a 2 year risk of around 50% would require the screening of 810,000 children. Over 750,000 children are born each year in Germany. If screening was recommended by the authorities and reimbursed by the payers (i.e. health authorities, insurance companies etc.), screening may reach over 50% of children. This scenario would provide a sufficient number of high-risk children with late-stage presymptomatic type 1 diabetes for one new trial of 3 years duration every 2 years. Moreover, such screening would identify a substantial number of children with undiagnosed stage 3 type 1 diabetes who would benefit by the prevention of diabetic ketoacidosis, as well as additional children with stage 1 type 1 diabetes who, with ongoing follow-up, will become eligible for clinical trials of late-stage presymptomatic type 1 diabetes. With the refinement provided by the progression likelihood score, it is possible to counsel families more appropriately regarding the actual risk of developing type 1 diabetes and to adjust the child’s follow-up accordingly, including the use of continuous glucose monitoring, which can help assess the child’s insulin requirement [37]. One consideration is that treatment with teplizumab may be introduced for the treatment of stage 2 type 1 diabetes in the future [2]. Therefore, future trials may need to be designed with teplizumab as the control arm. This may be challenging but is also likely to accelerate the search for safe and effective treatments. Finally, it is important that the screening age be aligned to the available treatment options. For example, teplizumab is currently only available for children over 8 years old. Because the greatest benefit and highest risk may occur at a younger age [6], it is important that alternative treatments that can be tested in younger children are made available.

Our screening and staging cost estimate for recruitment into a trial was over €17 million. This was achieved using an inexpensive initial screening test that limited the laboratory costs to less than €10 per screened child. This cost could be reduced further by raising the threshold for positivity in the screening ELISA test so that fewer samples require confirmation testing. Examination of the ELISA screening results in the 59 high-risk children (ESM Table 7) suggests that thresholds for positivity could be raised substantially without a loss in sensitivity. Screening may also be introduced locally and the development of point-of-care tests that detect high autoantibody titres should be considered. Notably, restricting trials to children with stage 2 type 1 diabetes would increase screening costs by almost 100%. The overall cost benefit will depend on how long the development of stage 3 type 1 diabetes can be delayed as well as the costs that might be saved by preventing diabetic ketoacidosis in previously undiagnosed children identified through screening. The cost of screening per child identified as having either undiagnosed diabetes or above a 50% risk of clinical diabetes within 2 years in Germany is approximately €33,400. This and the immunotherapy costs would need to be weighed against the costs incurred by several years of insulin therapy, which vary substantially between countries, as well as glucose monitoring activities and other potential reductions such as hospital admission. Of relevance to this, teplizumab delayed insulin therapy by 32 months in an earlier study [2].

In conclusion, we have modelled the feasibility and effectiveness of population-based islet autoantibody screening for identifying children who are expected to benefit from immunotherapy. We recommend that these findings are considered as evidence for extending presymptomatic type 1 diabetes screening guidelines.

## Supplementary information

ESM 1 (PDF 763 kb)

## Data Availability

The de-identified individual participant data that underlie the results (text, tables, figures and ESM, excluding genetic data) reported in this article can be shared between 9 and 36 months after publication of the article. Requests will be honoured from researchers who provide a methodologically sound proposal and who complete a Data Use Agreement with the Helmholtz Zentrum Muenchen. Requests should be directed by email to the corresponding author.

## Abbreviations

CPH: Cox proportional hazards
GADA: autoantibody
GAD: Glutamic acid decarboxylase
IA-2A: Insulinoma antigen-2 autoantibody
IAA: Insulin autoantibody
LR: Logistic regression
ZnT8A: Zinc transporter 8 autoantibody
