# Correction to: Progression likelihood score identifies substages of presymptomatic type 1 diabetes in childhood public health screening

**DOI:** 10.1007/s00125-022-05798-z

**Published:** 2022-10-04

**Authors:** Andreas Weiss, Jose Zapardiel-Gonzalo, Franziska Voss, Manja Jolink, Joanna Stock, Florian Haupt, Kerstin Kick, Tiziana Welzhofer, Anja Heublein, Christiane Winkler, Peter Achenbach, Anette-Gabriele Ziegler, Ezio Bonifacio, for the Fr1da-study group

**Affiliations:** 1 Institute of Diabetes Research, Helmholtz Munich, German Research Center for Environmental Health, Munich, Germany; 2 German Center for Diabetes Research (DZD), Munich, Germany; 3 Forschergruppe Diabetes e.V. at Helmholtz Zentrum München, Munich, Germany; 4 Technical University Munich, School of Medicine, Forschergruppe Diabetes at Klinikum rechts der Isar, Munich, Germany; 5 Center for Regenerative Therapies Dresden, Faculty of Medicine, Technische Universität Dresden, Dresden, Germany; 6 Paul Langerhans Institute Dresden of Helmholtz Centre Munich at University Clinic Carl Gustav Carus of TU Dresden, Faculty of Medicine, Dresden, Germany

**Correction to: Diabetologia**

10.1007/s00125-022-05780-9

In the Conclusions/interpretation section of the Abstract, the following, potentially misleading, text has been changed:

Original wording: ‘Public health screening for islet autoantibodies found 0.027% of children to have undiagnosed clinical type 1 diabetes and 0.038% to have undiagnosed presymptomatic type 1 diabetes. Of the latter group, our novel likelihood ratio score predicted approximately 50% to be at risk of developing clinical type 1 diabetes within 2 years.’

Modified wording: ‘Public health screening for islet autoantibodies found 0.027% of children to have undiagnosed clinical type 1 diabetes and 0.038% to have undiagnosed presymptomatic stage 2 or stage 1b type 1 diabetes, with 50% risk to develop clinical type 1 diabetes within 2 years.’

The original article has been corrected.

