# Supplementary material for: Progression likelihood score identifies substages of presymptomatic type 1 diabetes in childhood public health screening
Source: Diabetologia. 2022 Aug 27;65(12):2121–31. doi: 10.1007/s00125-022-05780-9 (PMC9630406; doi:10.1007/s00125-022-05780-9)
Supplement: Supplementary file 1 — (PDF 763 kb) [file 125_2022_5780_MOESM1_ESM.pdf]

Progression likelihood score identifies substages of presymptomatic type 1 diabetes in childhood public health screening

Andreas Weiss[1,2*], Jose Zapardiel-Gonzalo[1,2*], Franziska Voss[1*], Manja Jolink[1], Joanna Stock[1], Florian Haupt[1,2,4], Kerstin Kick[3], Tiziana Welzhofer[3], Anja Heublein[1], Christiane Winkler[1,2,4], Peter Achenbach[1,2,3,4], Anette-G. Ziegler[1,2,3,4#], Ezio Bonifacio[2,5,6#] for the Fr1da-study group

[1]Institute of Diabetes Research, Helmholtz Munich, German Research Center for Environmental Health, Munich, Germany.

[2]German Center for Diabetes Research (DZD), Munich, Germany

[3]Technical University Munich, School of Medicine, Forschergruppe Diabetes at Klinikum rechts der Isar, Munich, Germany.

[4]Forschergruppe Diabetes e.V. at Helmholtz Zentrum München, Munich, Germany

[5]Center for Regenerative Therapies Dresden, Faculty of Medicine, Technische Universität Dresden, Dresden, Germany

[6]Paul Langerhans Institute Dresden of Helmholtz Centre Munich at University Clinic Carl Gustav Carus of TU Dresden, Faculty of Medicine, Dresden, Germany

* Shared first authors

# Shared last authors

APPENDIX – ESM CONTENT

Table of contents:

ESM Table 1 2
ESM Table 2 6
ESM Table 3 5
ESM Table 4 7
ESM Table 5 8
ESM Table 6 9
ESM Table 7 10
ESM Figure 1 11
ESM Figure 2 12
ESM Figure 3 13
ESM Figure 4 14
ESM Figure 5 15
ESM Figure 6 156
ESM Figure 7 17

ESM Table 1: List of SNPs for the genetic risk score calculation

SNP	Gene, Allele, or Haplotype	Score weight for genotype or per allele
**HLA class II**		
rs17426593	*HLA DR4-DQ8/DR4-DQ8*	3.15
rs2187668		3.98
rs7454108	*HLA DR3/DR4-DQ8*	
rs3129889	*HLA DRB1*1501*	
rs1794265	*HLA DQB1*0503*	
**HLA class I**		
rs1264813	*HLA A 24*	0.43
rs2395029	*HLA B 5701*	0.92
**Non-HLA SNPs**		
rs2476601	*PTPN22*	0.76
rs2816316	*RGS1*	0.16
rs3024505	*IL10*	0.22
rs1990760	*IFIH1*	0.16
rs3087243	*CTLA4*	0.16
rs10517086	*C4orf52*	0.19
rs2069763	*IL2*	0.11
rs6897932	*IL7R*	0.19
rs3757247	*BACH2*	0.19
rs9388489	*C6orf173*	0.14
rs6920220	*TNFAIP3*	0.15
rs1738074	*TAGAP*	0.05
rs7804356	*SCAP2*	0.15
rs4948088	*COBL*	0.17
rs7020673	*GLIS3*	0.23
rs12722495	*IL2RA*	0.47
rs947474	*PRKCQ*	0.15
rs10509540	*RNLS/C10orf59*	0.25
rs689	*INS*	0.65
rs4763879	*CD69*	0.06
rs2292239	*ERBB3*	0.36
rs3184504	*SH2B3*	0.24
rs1465788	*ZFP36L1*	0.13
rs17574546	*RASGRP1*	0.13

rs3825932	*CTSH*	0.15
rs12708716	*CLEC16A*	0.15
rs4788084	*IL27*	0.20
rs7202877	*CTRB2*	0.19
rs2290400	*ORMDL3*	0.25
rs7221109	*CCR7*	0.15
rs45450798	*PTPN2*	0.09
rs763361	*CD226*	0.12
rs425105	*PRKD2*	0.21
rs2281808	*SIRPG*	0.07
rs3788013	*UBASH3a*	0.16
rs5753037	*RPS3AP51*	0.15
rs229541	*IL2B*	0.18
rs5979785	*TLR8*	0.09
rs2664170	*GAB3*	0.14

The risk score is calculated by multiplying the number of risk alleles (i. e. 0, 1 or 2 for each single SNP) with the weight assigned to each SNP and then summing up the weighted contributions of all SNPs plus an additive constant for each of the two HLA class II categories, 3.15 for children who have the HLA *DR4-DQ8/DR4-DQ8* genotype or 3.98 for children who have the HLA *DR3/DR4-DQ8* genotype.

As an example, the risk score for a child with HLA *DR4-DQ8/DR4-DQ8*, homozygous for the risk allele of rs1264813 (weight 0.43), heterozygous for the risk allele of rs2395029 (weight 0.92), homozygous for the non-risk allele of rs2476601 (weight 0.76) and for all other SNPs in the genetic risk score is calculated as follows:

Risk score = 3.15 + (2 * 0.43) + (1 * 0.92) + (0 * 0.76) + 0 = 4.93

ESM Table 2. Characteristics of children who did and did not participate in follow-up staging.

	N (%); Median (IQR)		
Variable	Staging completed (*n*=323)	Staging refused (*n*=83)	*p*-value*
Sex (male)	175 (54.2%)	47 (56.6%)	0.78
Age at screening (years)	4.1 (3.1–5.4)	4.2 (2.7−5.4)	0.70
First degree relative with T1D			
No	275 (85.1%)	69 (83.1%)	
Yes	48 (14.9%)	14 (16.9%)	0.77
Mother	15 (4.6%)	3 (3.6%)	
Other	33 (10.3%)	11 (13.3%)	
Number of autoantibodies			0.20
2	99 (30.7%)	27 (32.5%)	
3	111 (34.4%)	37 (44.6%)	
4	112 (34.7%)	18 (21.7%)	
Genetic risk score	12.8 (11.7–13.9)	12.8 (11.5–13.7)	0.53
BMI z-score	0.22 (-0.39–0.92)	0.58 (-0.19–1.30)	0.02

BMI, Body Mass Index; T1D, type 1 diabetes

*p-values were taken from binomial tests, chi squared-tests and Mann-Whitney-U-tests respective to the variables

ESM Table 3. Univariable Cox proportional hazards models for predicting the progression from stage 1 to stage 3 type 1 diabetes

		N	HR (95% CI)	*P* value
First-degree relative[A]				
	No	250		Reference
	Yes	43	0.93 (0.44–1.97)	0.86
Sex	Female	137		Reference
	Male	156	0.83 (0.50–1.37)	0.47
Staging age		293	1.08 (0.93–1.26)	0.30
Participation in intervention[B]				
	No	144		Reference
	Yes	149	0.78 (0.47–1.28)	0.33
HbA_{1c} (+0.74 mmol/l [0.1%])[C]		290	1.20 (1.10–1.31)	<0.001
BMI[D]	Normal	216		Reference
	Overweight	52	1.97 (1.06–3.64)	0.031
	Obesity	23	2.73 (1.31–5.69)	0.007
Blood glucose at OGTT				
	0 min (+0.555 mmol/l [10 mg/dl])[C]	292	0.82 (0.66–1.02)	0.069
	30 min (+0.555 mmol/l [10 mg/dl])[C]	289	0.97 (0.90–1.05)	0.47
	60 min (+0.555 mmol/l [10 mg/dl])[C]	290	1.16 (1.05–1.27)	0.003
	90 min (+0.555 mmol/l [10 mg/dl])[C]	289	1.22 (1.10–1.35)	<0.001
	120 min (+0.555 mmol/l [10 mg/dl])[C]	293	1.18 (1.01–1.37)	0.032
HLA genotype				
	DR3/DR4-DQ8	55	1.80 (1.05–3.10)	0.034
	other	188		Reference
Genetic Risk Score (continuous)		241	1.13 (0.96–1.33)	0.13
Genetic Risk Score (category)				
	1st Tertile	81		Reference
	2nd Tertile	80	1.57 (0.79–3.13)	0.20
	3rd Tertile	80	1.78 (0.92–3.44)	0.087
Islet Autoantibodies				
	2	93		Reference
	3 or 4	199	1.88 (1.04–3.42)	0.038
IAA	Negative	45		Reference
	Positive	247	0.79 (0.41–1.52)	0.48
IAA titer (continuous)		292	1.00 (0.98–1.01)	0.48
GADA	Negative	32		Reference
	Positive	261	0.68 (0.33–1.37)	0.28
GADA titer (continuous)		293	1.00 (1.00–1.00)	0.66
IA-2A	Negative	113		Reference
	Positive	180	3.60 (1.91–6.78)	<0.001
IA-2A titer (continuous)		293	1.003 (1.002–1.004)	<0.001
IA-2A category (ordinal[E])			1.89 (1.51–2.37)	<0.001
ZnT8A	Negative	97		Reference
	Positive	196	1.64 (0.90–2.98)	0.11
ZnT8A titre (continuous)		293	1.00 (1.00–1.00)	0.18

[A] First-degree relative of a participant with type 1 diabetes
[B] Mechanistic insulin intervention trial
[C] Increments are given in parentheses

[D]Standardized body mass index (BMI) was calculated using the World Health Organization Child Growth Standards based on height and weight and age [15].
[E] IA-2A as negative and tertiles of IA-2A titres: 3–100 arbitrary units, 100–290 arbitrary units, >290 arbitrary units

HR, hazard ratio; CI, confidence interval; FDR, first-degree relative; HbA_{1c}, haemoglobin A1c; BMI, body mass index; OGTT, oral glucose tolerance test; HLA, human leukocyte antigen; IAA, insulin autoantibody; GADA, glutamic acid decarboxylase autoantibody; IA-2A, islet antigen-2 autoantibody; ZnT8A, zinc transporter 8 autoantibody

ESM Table 4. Logistic regression analysis calculated using 202 samples with valid data for the factors associated with progression from stage 1 to stage 3 type 1 diabetes (using normalized variables)

	**OR (95% CI)**	**p-value**
Intercept	0.05 (0.02–0.10)	<0.001
OGTT 90 min glucose	2.21 (1.38–3.75)	0.002
IA-2A titer	2.41 (1.50–4.0)	<0.001
HbA_{1c}	1.95 (1.08–3.92)	0.040

OR, odds ratio; CI, confidence interval; OGTT, oral glucose tolerance test; IA-2A, islet antigen-2 autoantibody; HbA_{1c}, haemoglobin A_{1c}

ESM Table 5. Bias and standard error of 100,000 bootstrap replicates of the 2-year risk for progression from stage 1b to stage 3 type 1 diabetes, calculated using the Cox proportional hazards analysis and logistic regression risk formulas

	**2-year risk**	**Bootstrap bias**	**Bootstrap standard error**
**Cox proportional hazards**	46%	-3%	12%
**Logistic regression**	45%	3%	11%

ESM Table 6. Two-year risk of progression from stage 1 to stage 3 type 1 diabetes and sensitivity for thresholds per 10th centile increase of each factor and for the score derived from the Cox proportional hazards analysis of the combination of HbA_{1c} and IA-2A expressed as an ordinal scale

	**OGTT 90 minute glucose**		**HbA_{1c}**		**IA-2A titer category**		**CPH score from HbA_{1c} and IA-2A**	
**Percentile**	**2-year risk (%)**	**Sensitivity (%)**	**2-year risk (%)**	**Sensitivity (%)**	**2-year risk (%)**	**Sensitivity (%)**	**2-years risk (%)**	**Sensitivity (%)**
0	8	100	8	100	8	100	8	100
10	9	100	9	100	13	100	9	100
20	10	95	11	95	13	100	10	95
30	11	95	12	90	13	100	11	95
40	13	95	12	70	16	95	13	90
50	14	80	12	70	16	95	16	90
60	16	75	13	55	21	80	21	90
70	18	60	15	45	21	80	22	75
80	19	45	15	30	21	50	28	60
90	20	25	17	20	21	50	37	30

OGTT, oral glucose tolerance test; HbA_{1c}, haemoglobin A1c; IA-2A, islet antigen-2 autoantibody; CPH, Cox proportional hazards

ESM Table 7. Characteristics of the 29 children with stage 1b type 1 diabetes with a Cox proportional hazards score > 4.0 (90th centile)

Age at staging (years)	3Screen-ELISA (AU)	IAA positive	GADA positive	IA-2A positive	ZnT8A positive	IA-2A titer (AU)	IA-2A category	Blood glucose (mmol/l) at OGTT 90 min	HbA_{1c} (mmol/mol)	CPH score	Follow-up (months)	T1D progression
6.10	>2000	no	no	yes	yes	259.1	2	10.0	46.5	28.73	17.60	yes
5.73	>2000	yes	yes	yes	yes	298.4	3	11.0	33.3	17.60	4.08	yes
1.62	>2000	yes	yes	yes	yes	352.9	3	8.9	38.8	16.01	34.31	yes
3.39	>2000	yes	yes	yes	yes	538.5	3	9.9	33.3	11.92	4.44	yes
2.51	>2000	yes	yes	yes	no	487.8	3	7.9	38.8	11.06	15.30	yes
3.22	1195	yes	yes	yes	yes	343.5	3	7.9	35.5	7.59	49.18	yes
3.47	>2000	no	yes	yes	yes	355.8	3	7.5	36.6	7.51	7.86	yes
4.11	>2000	yes	yes	yes	yes	317.4	3	7.7	35.5	7.03	1.68	no
5.58	>2000	yes	yes	yes	yes	155.0	2	9.1	36.6	6.81	35.95	yes
10.17	1386	yes	yes	yes	yes	291.5	3	7.6	34.4	6.08	48.88	yes
6.47	>2000	yes	no	yes	yes	331.6	3	7.8	33.3	5.69	25.86	yes
5.67	>2000	yes	yes	yes	yes	495.3	3	7.0	35.5	5.67	25.53	no
2.61	>2000	no	yes	yes	yes	326.2	3	6.6	36.6	5.50	4.21	no
4.32	453	yes	yes	yes	yes	373.7	3	6.2	37.7	5.44	23.45	yes
2.19	>2000	yes	yes	yes	yes	324.0	3	6.9	35.5	5.35	8.68	yes
2.24	464	yes	yes	yes	yes	338.8	3	6.9	35.5	5.35	11.18	yes
2.38	1318	yes	yes	yes	yes	285.4	2	8.7	35.5	5.25	11.61	no
3.29	>2000	yes	yes	yes	no	383.5	3	7.2	34.4	5.20	11.45	no
10.81	>2000	no	yes	yes	yes	290.9	3	6.4	36.6	5.09	38.45	no
4.33	>2000	yes	no	yes	yes	332.9	3	7.0	34.4	5.00	12.60	no
2.99	>2000	yes	yes	yes	no	204.2	2	7.0	39.9	4.83	47.86	no
6.70	>2000	yes	yes	yes	yes	419.8	3	6.9	34.4	4.81	0.03	no
2.10	>2000	no	no	yes	yes	335.3	3	7.6	32.2	4.73	14.51	yes
2.32	1949	yes	no	yes	yes	331.1	3	6.4	35.5	4.49	26.58	yes
4.45	>2000	yes	yes	yes	yes	213.1	2	7.5	37.7	4.48	35.33	yes
2.68	1902	yes	yes	yes	yes	323.2	3	6.3	35.5	4.40	11.51	no
2.90	496	no	no	yes	yes	112.1	2	8.2	35.5	4.40	50.23	yes
4.73	>2000	yes	yes	yes	yes	203.6	2	7.4	37.7	4.31	5.89	yes
5.22	>2000	yes	yes	yes	yes	242.4	2	8.4	34.4	4.12	11.28	yes

ELISA, enzyme-linked immunosorbent assay; AU, arbitrary units; IAA, insulin autoantibody; GADA, glutamic acid decarboxylase autoantibody; IA-2A, islet antigen-2 autoantibody; ZnT8A, zinc transporter 8 autoantibody; OGTT, oral glucose tolerance test; HbA_{1c}, haemoglobin A_{1c}; CPH, Cox proportional hazards; T1D, type 1 diabetes

IA-2A category as negative (0) and tertiles of IA-2A titres: 3–100 arbitrary units (1), 100–290 arbitrary units (2), >290 arbitrary units (3)

ESM Figure 1. Cumulative risk of progression to stage 3 type 1 diabetes in children with stage 2 type 1 diabetes at initial staging or during follow-up.
Shading indicates the 95% confidence intervals. The follow-up starts from the time of diagnosis of stage 2 type 1 diabetes. The numbers of children at risk are indicated below each time-point.

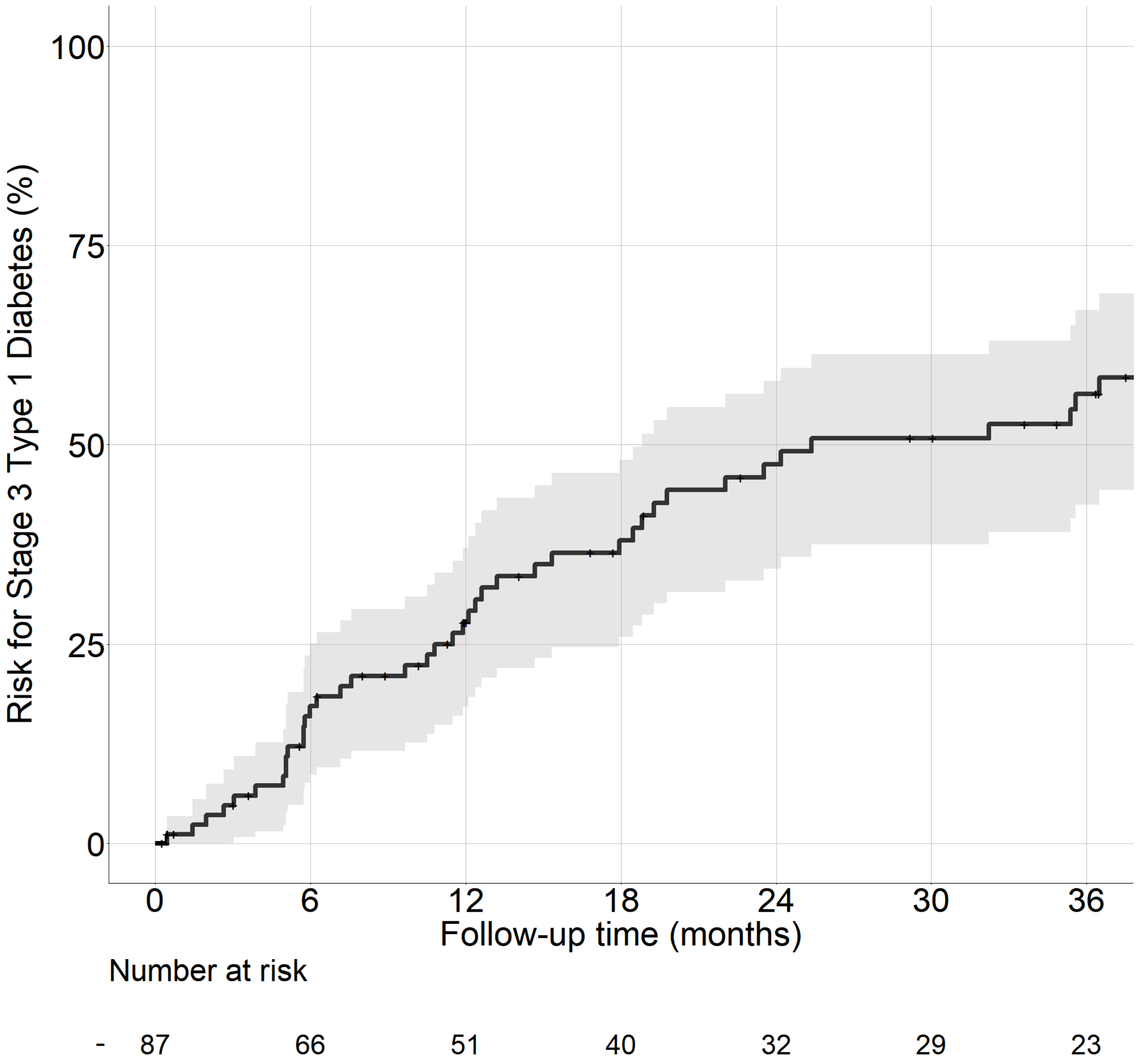

ESM Figure 2. Importance of each variable in the penalised logistic regression (panel A) and random forest (panel B) analyses.

Data available for all variables from 191 children who were followed for at least two years or had developed stage 3 type 1 diabetes prior to two years follow-up were used to build the full model. The variance importance was based on the absolute value of the corresponding t-statistic in logistic regression and computed from the permuted out-of-bag data in the random forest analysis.
AUC, area under the curve; FDR, first-degree relative; GADA, glutamic acid decarboxylase autoantibody; HbA_{1c}, haemoglobin A1c; HLA, human leukocyte antigen; IA-2A, islet antigen-2 autoantibody; IAA, insulin autoantibody; OGTT, oral glucose tolerance test; ZnT8A, zinc transporter 8 autoantibody;

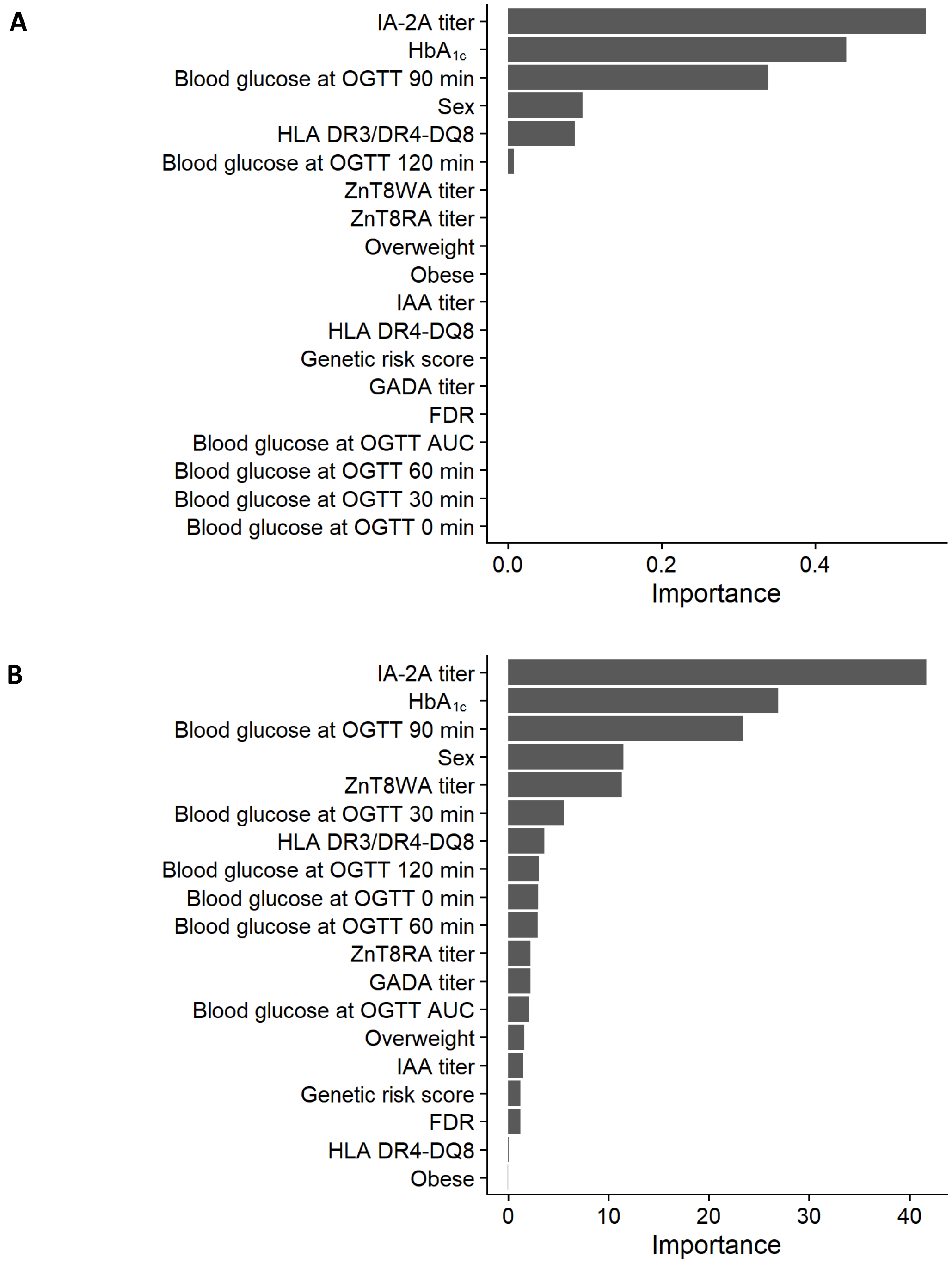

ESM Figure 3. Two-year risk of progression from stage 1 to stage 3 type 1 diabetes (black) and the sensitivity (frequency of children who developed stage 3 type 1 diabetes within 2 years; red) at the 10th centiles of the risk score calculated by logistic regression.

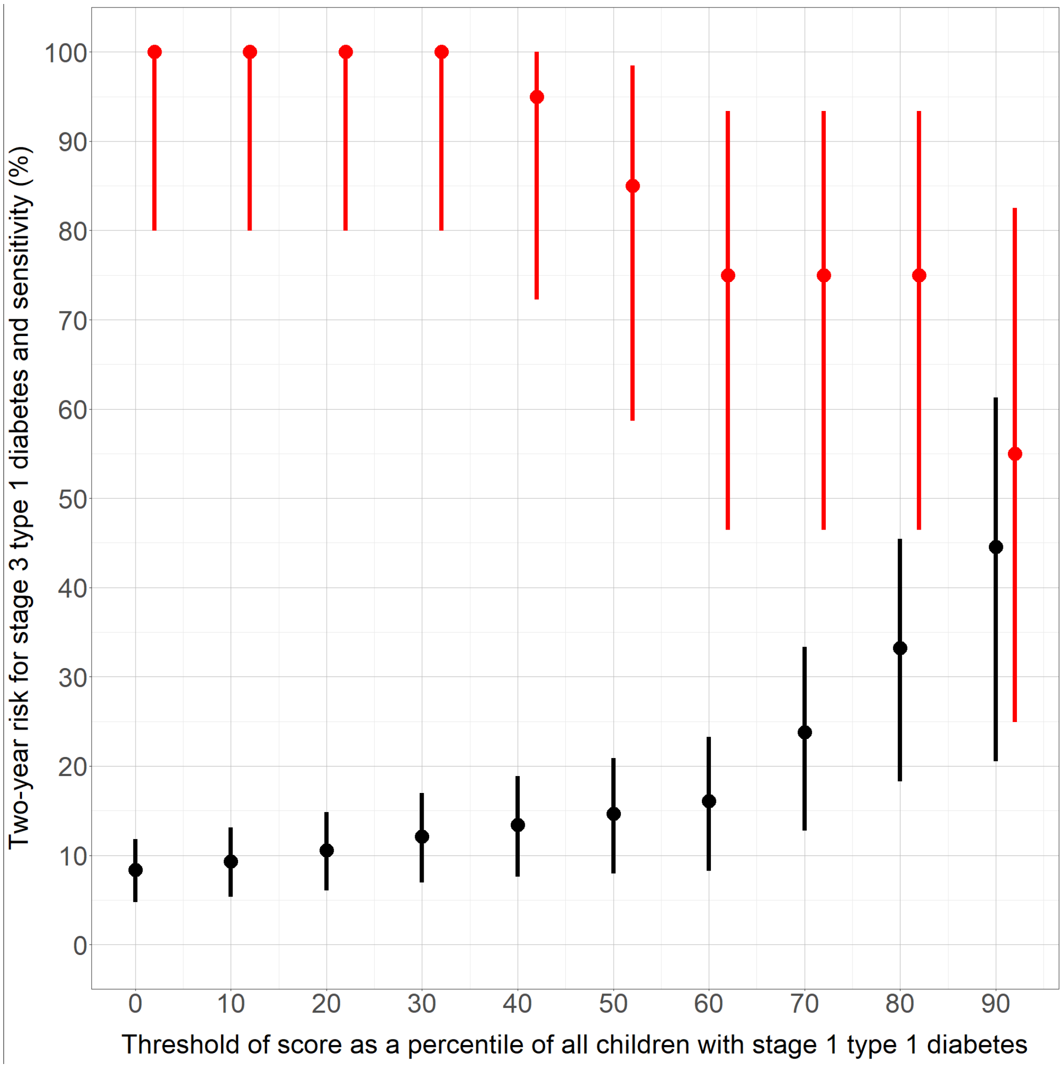

ESM Figure 4. Risk of progression to stage 3 type 1 diabetes for children with a logistic regression progression likelihood risk score above (blue) and below (red) the 90th centile.

Shading indicates the 95% confidence intervals. Analyses were performed using 287 children with complete data for all three variables included in the risk score (glucose at 90 minutes in oral glucose tolerance tests, islet antigen-2 autoantibody titre category, and haemoglobin A1c). The numbers of children at risk are indicated below each time-point.

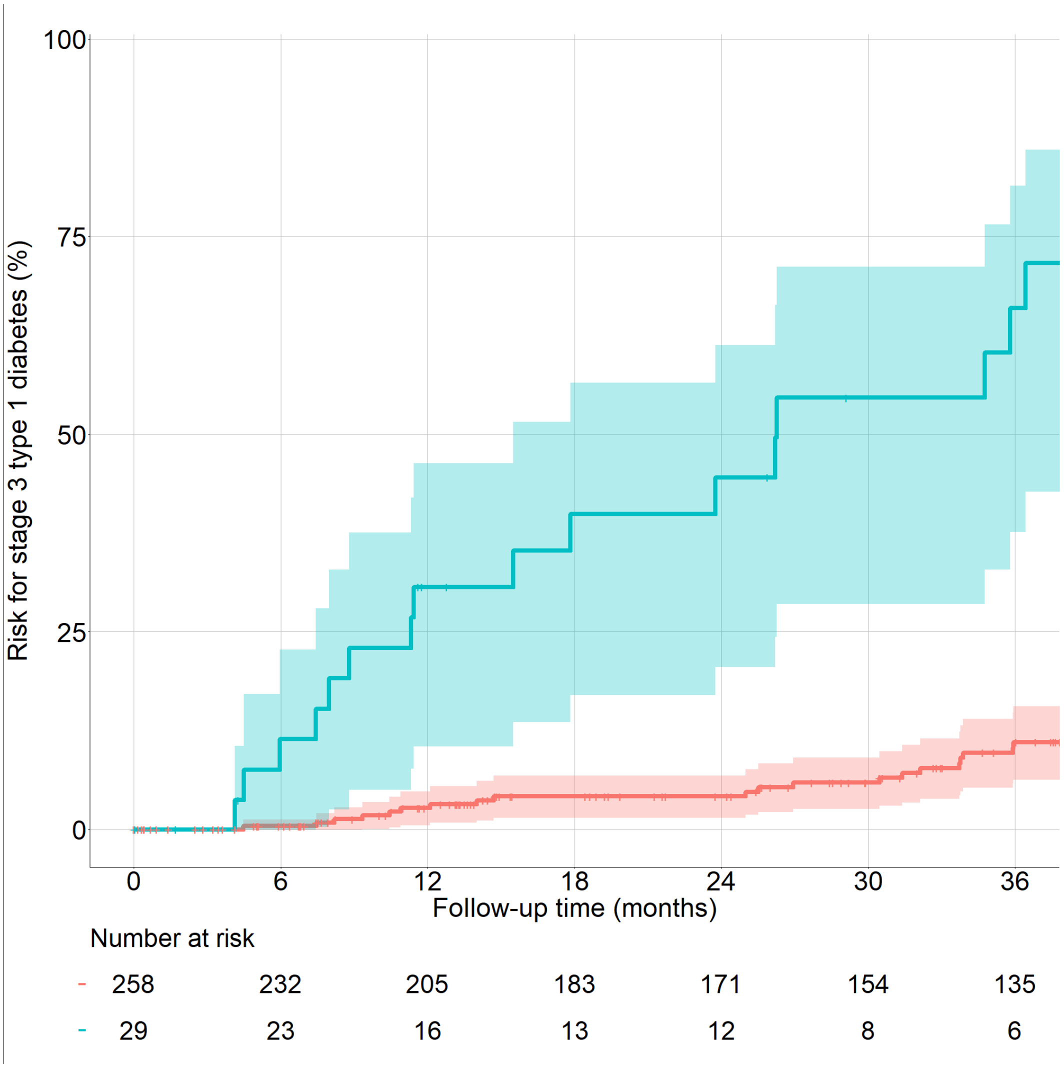

ESM Figure 5. Progression to stage 3 type 1 diabetes in relation to the Cox proportional hazards (CPH) progression likelihood score for a validation cohort of 46 multiple islet autoantibody children who are first-degree relatives of participants with type 1 diabetes.

The *y* axis indicates the time (months) to onset of stage 3 type 1 diabetes (filled circles) or the time to last visit in children without type 1 diabetes (open circles). The follow-up time was calculated from the first sample where the glucose at 90 minutes in oral glucose tolerance tests, islet antigen-2 autoantibody titre category, and haemoglobin A1c data were available to calculate the CPH progression likelihood score (*x* axis). The vertical dashed line represents a score of 4, which corresponds to the threshold for classification as stage 1a type 1 diabetes (red circles) and stage 1b type 1 diabetes (blue circles).

CPH, Cox proportional hazards.

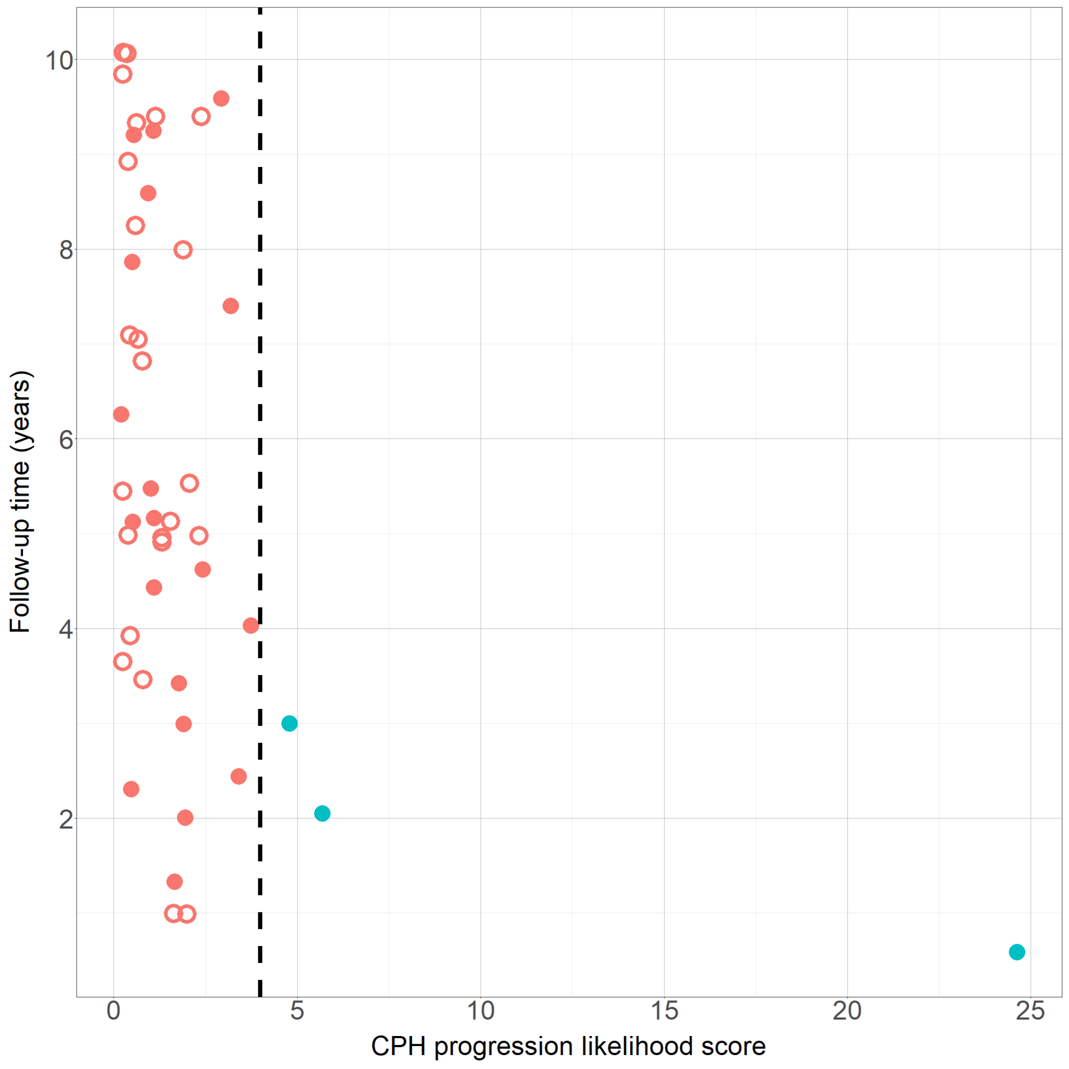

ESM Figure 6. Risk of progression to stage 3 type 1 diabetes for children with stage 1a type 1 diabetes who developed a progression likelihood risk score above the 90th centile (stage 1b type 1 diabetes) on follow-up.

The progression likelihood score was determined in follow-up visits for children with stage 1a type 1 diabetes. Twenty-six developed scores > 4.0 (stage 1b type 1 diabetes) in the absence of stage 2 or stage 3 type 1 diabetes. Progression to stage 3 type 1 diabetes is shown for the 26 children. The follow-up time was calculated from the first sample where the CPH progression likelihood score was > 4.0. Shaded areas represent the 95% CI. The numbers of children at risk are indicated below each time-point.

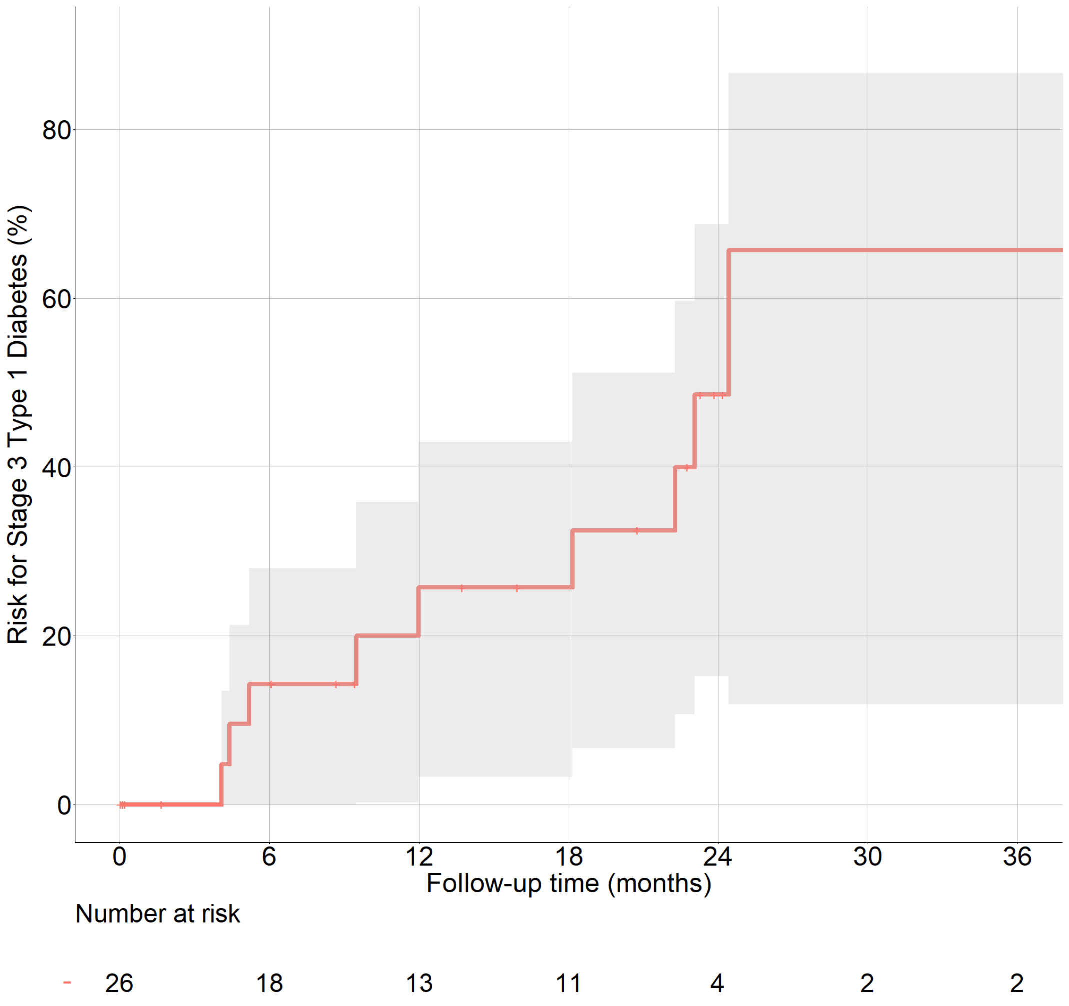

ESM Figure 7 Risk of progression to stage 3 type 1 diabetes for children who were not in the intervention arm of the Fr1da study using the CPH progression likelihood score below (stage 1a, red) and above (stage 1b, blue) a score of 4.

Shading indicates the 95% confidence intervals. Analyses were performed using 139 children with complete data for all three variables included in the risk score (glucose at 90 minutes in oral glucose tolerance tests, islet antigen-2 autoantibody titre category, and haemoglobin A1c). Two-year progression risks were 4.2% (95% CI, 0.1-8.1%) in children classified as stage 1a and 48.7% (95% CI, 1.1 -70.4%) in children classified as stage 1b (p = 0.002). The numbers of children at risk are indicated below each time-point.

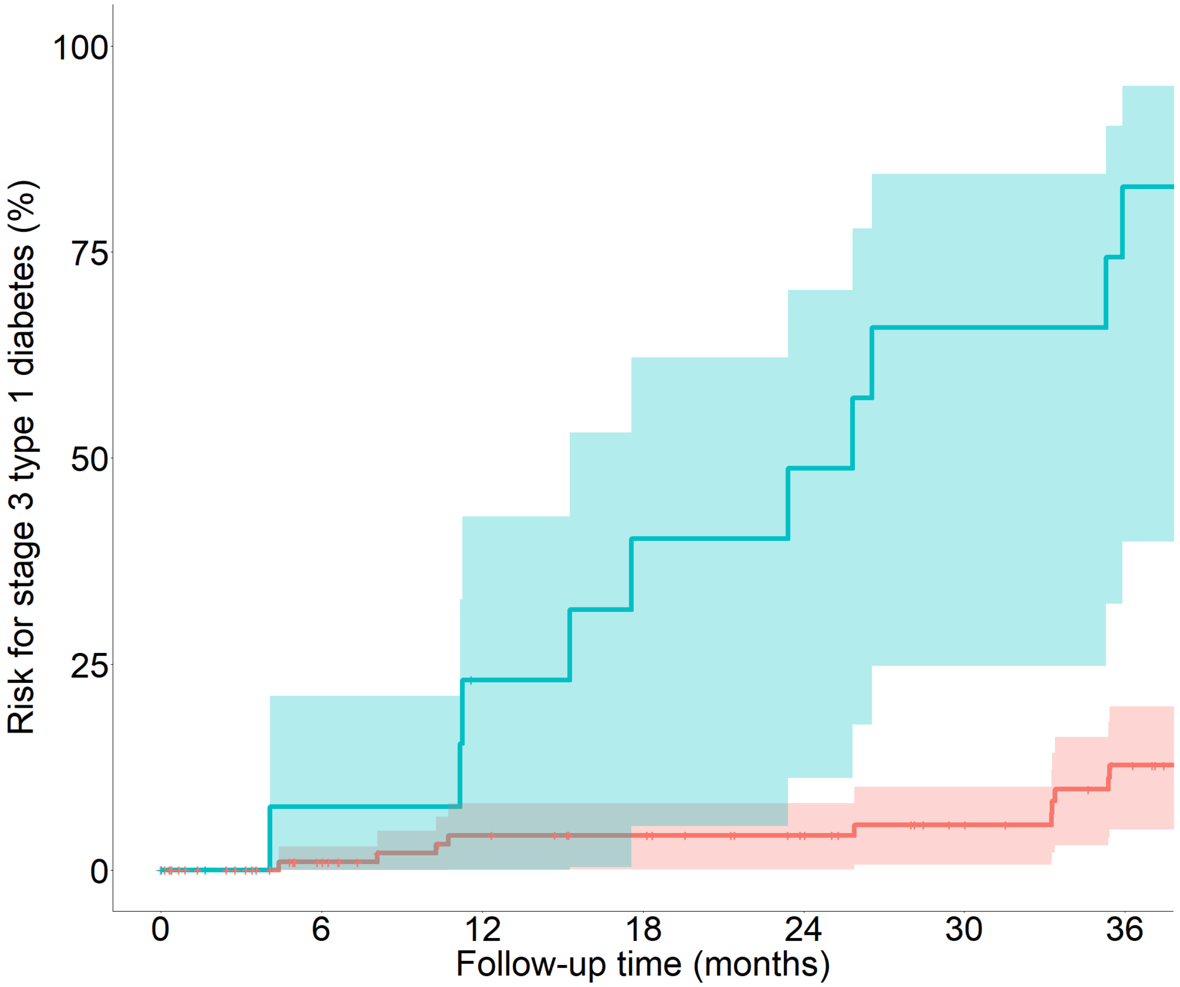

Number at risk

	0	6	12	18	24	30	36
-	124	98	88	84	76	68	59
-	15	12	9	7	6	4	2
